# Cellular immune responses in peripheral blood lymphocytes of *Giardia* infected squirrel monkey (*Saimiri boliviensis boliviensis*) treated with Fenbendazole

**DOI:** 10.1371/journal.pone.0198497

**Published:** 2018-11-09

**Authors:** Pramod N. Nehete, Gregory Wilkerson, Bharti P. Nehete, Sriram Chitta, Julio C. Ruiz, Henrieta Scholtzova, Lawrence E. Williams, Christian R. Abee, John A. Vanchiere

**Affiliations:** 1 Department of Veterinary Sciences, The University of Texas MD Anderson Cancer Center, Bastrop, Texas, United States of America; 2 The University of Texas Graduate School of Biomedical Sciences at Houston, Houston, Texas, United States of America; 3 New York University School of Medicine, Department of Neurology, New York,NY, United States of America; 4 Louisiana State University, Health Science Center, Section of Pediatric Infectious Diseases, Shreveport, Louisiana, United States of America; Centre de Recherche en Cancerologie de Lyon, FRANCE

## Abstract

Cellular immune responses were tested to determine the effect of fenbendazole on the function of lymphocytes from Bolivian squirrel monkeys (*Samiri boliviensis boliviensis*). *Giardia*-infected squirrel monkeys were treated with commercially available fenbendazole (FBZ)-medicated monkey chow. Immune responses were compared between historical controls (*Giardia naïve*, untreated with FBZ (control animals)) and *Giardia*-infected, FBZ-treated squirrel monkeys (study animals). Peripheral blood lymphocytes from *study* monkeys had significantly lower stimulation indices compared to control animals when cultured *in vitro* with concanavalin A (Con A) (p<0.0001), phytohaemagglutinin (PHA) (p<0.0001) and lipopolysaccharide (LPS) (p<0.0001). PBMCs were also analyzed for IFN-γ producing cells in response to stimulation with Con A, PHA, PWM, and LPS by the cytokine ELISPOT assay. Significantly higher responses to Con A- (p<0.0001), and PHA- (p<0.001) stimulated cultures from *Giardia-infected* and fenbendazole treated compared to controls. Flow cytometric analysis for expression of cell surface markers revealed a significant increase in B- and NKT-lymphocytes and significant decrease in CD14+CD16+ monocytes after FBZ treatment. Also, circulating plasma cytokines IFN-γ, TNF-α, IL-12p40, IL-1β, IL-10, IL-13, IL-1ra, IL-6 and IL-4 were significantly decreased after FBZ treatment. Comparison of hematologic parameters between controls and FBZ-treated squirrel monkeys revealed significantly lower numbers of total leukocytes, neutrophils, monocytes, and eosinophils compared to controls. However, erythrocyte indices (red cell count, hemoglobin and hematocrit were significantly higher in FBZ-treated monkeys. Our findings suggest that fenbendazole treatment may alter sensitive immune and molecular measures of inflammation. Postponing the experimental use of squirrel monkeys until at least 6 weeks after FBZ treatment should be considered.

## Introduction

The genus *Giardia* consists of protozoan parasites known to infect a wide range of amphibian, reptilian, avian, and mammalian hosts. All *Giardia* isolated in mammals are currently classified as *Giardia lamblia* (synonyms, *G*. *duodenalis* and *G*. *intestinalis*) [1, 2]. Despite the availability of efficacious antiparasitic drugs, *Giardia*-infections (Giardiasis) are currently considered to be a re-emerging parasitic disease of humans and some animals. In humans, the disease is especially prevalent among children living in environments where personal hygiene standards are poor [1]. In animals, the prevalence of Giardiasis is likewise noted to be highest among young animals housed under suboptimal conditions, around 10% in adult domestic dogs, in 50% of pups kept as companion animals, and in nearly 100% of dogs housed at some breeding kennels [3, 4].

Fenbendazole (FBZ) is a highly efficacious broad-spectrum anthelmintic drug that is used for treatment of numerous helminth and protozoan intestinal parasites including *Giardia*, of domesticated and laboratory animals [5, 6]. Its pharmacokinetic behavior, ovicidal activity, and wide safety margin in rats and mice make it an attractive choice for pinworm treatment. [2, 5, 7, 8]. In dogs, it is effective against most intestinal helminths [5]. FBZ is also effective for treatment of *Giardia*, lungworms, and flukes, but higher doses are needed for treatment of helminths and failure rates as high as 50% [5]. Many animal research facilities have been confronted with infestations of pinworms, a highly contagious parasite, which requires treating entire colonies and /or groups of animals as well as formication of entire research facilities for eradication. FBZ typically is administered to laboratory animals species through commercially available FBZ medicated (600 ppm) lab diets to reach a target dose of 8 to 12 ppm daily [6, 9].

*Giardia* infestation in laboratory animals is a significant problem in many research facilities. Although FBZ is very effective in the eradication of most intestinal helminth parasites, concerns about its potential effect on experimental results have not been investigated. It is believed that FBZ disrupts DNA, topoisomerase and nucleic acid synthesis in bacteria [10–12]. Effect of FBZ has been reported in studies of the immune system of sheep [2], and mice [13] and enhanced lipopolysaccharide-induced inflammation has been attributed to FBZ in rats [14]. Although no toxic effects have been reported from the use of FBZ at therapeutic levels, off target action of FBZ could alter or interfere with ongoing research experiments [15, 16]. There are many unanswered questions about whether FBZ affects rapidly dividing cells and whether particular organ systems such as the immune system would be affected negatively.

No research has been reported in biomedical literature regarding the effect of FBZ on the primate immune system. In the current study, we examine *Giardia-*infested squirrel monkeys for changes in routine immunologic parameters and assessors of immune function after treatment with a commercially available FBZ medicated (600 ppm) diet. The aim of this study was to determine whether FBZ had detrimental effects on the immune system of squirrel monkeys. These studies will promote understanding of adverse effects of FBZ treatment on the immune system in squirrel monkey. Importantly, our study will help veterinarians and researchers evaluate when experimental animals can be treated with FBZ and to understand when animals undergoing FBZ treatment might be assayed without the potential confounding effects on the immune system.

## Materials and methods

### Monkeys, care and housing

Biological samples for this study were derived from some of the approximately 400 Bolivian squirrel monkeys (*Saimiri boliviensis boliviensis)* in the Squirrel Monkey Breeding Research Resource (SMBRR) located at the University of Texas MD Anderson Cancer Center (UTMDACC) Michale E. Keeling Center for Comparative Medicine and Research (KCCMR) in Bastrop, TX. Animals within the SMBRR are maintained in social-breeding groups consisting of one adult male and 8–12 adult females with varying numbers of juveniles. Social breeding group are housed indoors in two connecting pens that are each 4’ wide x 6’ tall x 14’ long. The room containing the pens is maintained on a 12:12-h light: dark cycle, in a temperature range of 19.4 to 23.3°C, and humidity range of 30% to 70%. In addition to social interaction, the animals are provided with destructible enrichment manipulanda and different travel/perching materials on a rotating basis to promote species-typical behavior. Animals were observed daily and clinically evaluated by the clinical veterinarian as part of their routine colony maintenance protocol.

In the late part of 2017 *Giardia* was detected via light microscopy in the pooled fecal samples of all social groups of monkeys within the SMBRR. The presence of *Giardia* was also confirmed on individual squirrel monkeys using a Rapid Membrane Enzyme Immunoassay kit (*Giardia*/Cryptosporidium Quick Check, TechLab, Blacksburg, VA, USA).The SMBRR has been housed at the KCCMR since 2008 and *Giardia* had never been identified within any of yearly colony surveillance samples prior to this outbreak. The origin of the *Giardia*-infection in this colony is unknown but may have been derived from either contaminated produce or through the introduction of outside animals that were brought into the colony to diversify the genetics of the breeding stock.

### Ethics statement

This research was conducted at the AAALAC-I accredited KCCMR. All animal experiments were carried out according to the provisions of the Animal Welfare Act, PHS Animal Welfare Policy, and the principles of the NIH Guide for the Care and Use of Laboratory Animals. All procedures with animals were approved by the UTMDACC Institutional Animal Care and Use Committee.

### Diet

Squirrel monkeys housed at the SMBRR have *ad libitum* access to New World Primate Diet (Purina #5040) and filtered reverse-osmosis water. In addition, the animals are fed either fresh fruits or vegetables daily. Specialty foods, such as seeds, peanuts, raisins, yogurt, cereals, frozen juice cups and peanut butter, are also distributed daily as enrichment items. At no time were the subjects of this study deprived of food or water. Following the discovery of the *Giardia* outbreak within the colony, the squirrel monkeys were placed on FBZ-medicated diet (Lab Diet with a FBZ concentration of 600ppm) for 5 consecutive days. The animals were then returned to the SMBRR standard diet for a total of 4 weeks. After this four week period the animals were treated a second time with the medicated diet. During this second treatment regimen, animals were provided with the medicated diet on alternate days over a one week time period for a total of three additional FBZ-treatment days. FBZ medicated diet was stopped and study animals were returned to the SMBRR standard diet. After four week period of standard diet, blood was collected and analyzed at week 2, week 6 and week 14.

### Study groups

The study consisted of two study groups. The control group consisted of untreated, *Giardia*-negative animals (FBZ (-) Giardia (-)) group. The second group consisted of FBZ-treated *Giardia*-infected animals (FBZ (+) *Giardia* (+)). The Control group (FBZ (-) Giardia (-) consisted of 15, 3–5 year old female squirrel monkeys that had been randomly selected from the colony for blood screening in 2015, prior to the identification of Giardiasis in the colony. All subjects included in the study were healthy and in their normal social groups at the time they were sampled. Blood samples for all analyses in this study were collected from a peripheral vein into EDTA-coated collection tubes. All immune assays were performed by same procedures and reagents on freshly isolated PBMCs from control animals (FBZ (-) *Giardia* (-)) group as well as FBZ (+) *Giardia* (+) animals.

### Complete blood count and blood chemistry analysis

Blood samples were analyzed for a complete blood count (Siemens Advia 120 Hematology Analyzer, Tarrytown, NY). Parameters included: total WBC, total RBC, hemoglobin, hematocrit, RBC indices, WBC differential counts, and platelet count. Comparisons were made between each of the post-FBZ treatment samples and the control samples using a Dunnett’s test for multiple comparisons. In order to correct for using the control data in multiple comparisons only differences with a probability of less than 0.01 were considered to be significant.

### Blood collection and sample preparation

Approximately 2–3 ml of each blood sample was collected in EDTA coated collection tubes and immediately plasma was separated by centrifugation and stored at -80C until further use. Peripheral blood mononuclear cells (PBMCs) were isolated by ficoll-hypaque density gradient separation as described previously [17, 18]. Erythrocytes were removed by osmotic lysis in ACK lysing buffer (Life Technologies, Grand Island, NY), and the remaining nucleated cells were washed twice with RPMI supplemented with 10% fetal bovine serum (FBS) and used for immune assays.

### Flow cytometry

Cell-surface markers were determined using the following fluorescence labeled monoclonal antibodies specific to different lymphocytes subsets: T cells (CD3 PerCP, clone SP34-2, BD Pharmingen), B cells (CD20 APC clone L27, BD Pharmingen, San Jose, CA), monocytes (CD14 FITC clone M5E2 BioLegend, San Diego CA), and NK cells (CD16 PE clone 3G8, BD Pharmingen, San Jose, CA). Phenotype analysis of lymphocytes in peripheral blood from the monkeys was performed by surface staining of whole blood samples as described previously [18]. Briefly, 100μL of EDTA-preserved whole blood from each sample was added to individual 12mm×75mm polystyrene test tubes (Falcon, Lincoln Park, NJ, USA) containing pre-added monoclonal antibodies against CD3, CD14, CD16 and CD20 and incubated for 15 min at room temperature in the dark. After removing the red blood cells by incubating with FACS lysing solution (Becton Dickinson, USA), the mononuclear cells were washed twice with phosphate-buffered saline (PBS) containing FBS (0.5%) and sodium azide (0.1%) and re-suspended in 1% formaldehyde. The stained cells were acquired with FACSCalibur (Becton Dickinson, CA, USA) equipped with a 488 nm argon ion laser and a 635 nm red diode laser. The T, B, monocyte, NK and NKT cells from lymphocytes were gated on forward scatter versus side scatter dot plot and analysed using FlowJo software (Tree Star, Inc., Ashland, OR). Both compensation and isotypes controls were utilized to define specificity. All antibodies used in this study are cross reactive to *Saimiri boliviensis* as reported [19] and also in NIH Nonhuman Primate Reagent Resource core facility (http://www.NHPreagents.org).

### In vitro mitogen stimulation

PBMCs, freshly prepared from whole blood, were more than 90% viable, as determined by the trypan blue exclusion method, and for each immune assay, 10^5^ cells/well were used. The proliferation of PBMCs was determined by the standard MTT dye reduction assay, as previously described [19–22]. Briefly, aliquots of PBMCs (10^5^/well) were seeded in triplicate wells of 96-well, flat-bottom plates and individually stimulated for 72 h with the mitogens phytohemagglutinin (PHA), concanavalin-A (Con A), lipopolysaccharide (LPS), and pokeweed mitogen (PWM) (Sigma, St Louis, MO), each at a final concentration of 2 µg/mL. The culture medium without added mitogens served as a negative control. After culture for 72 h at 37°C in 5% CO_2_, 150uL of medium was removed from each well, and further incubated for 4 hr. with 15 µL of freshly prepared filtered MTT dye (5 mg/mL in PBS). After incubation, 175 µL of 0.04N hydrochloric acid in isopropanol (Sigma) was added and incubated for 30 min at room temperature for color development, before being read by an ELISA plate reader using a 490-nm filter (Victor, PerkinElmer, Shelton, CT). Results are expressed as stimulation index (SI) over the media control. Reported values are the mean of 3 replicates. The concentration of mitogen, number of PBMCs, and incubation time were standardized in our laboratory as optimal for stimulation of PBMCs isolated from healthy animals.

### ELISPOT assay for detecting antigen-specific IFN-γ producing cells

Freshly-isolated PBMCs as described above, were stimulated with the mitogens PHA, Con A, LPS and PWM (each at 2 μg/mL final concentration) to determine the numbers of IFN-γ-producing cells by the Enzyme Linked Immuno Spot (ELISPOT) assay using the methodology reported previously [19, 23, 24]. Briefly, aliquots of PBMCs (10^5^/well) were seeded in duplicate wells of 96-well plates (polyvinylidene difluoride backed plates, MAIP S 45, Millipore, Bedford, MA) pre-coated with the primary IFN-γ antibody and the lymphocytes were stimulated with the different mitogens. After incubation for 18–20 hr. at 37°C, the cells were removed and the wells were thoroughly washed with PBS and developed as per protocol provided by the manufacturer. Blue-Purple colored spots representing individual cells secreting IFN-γ were counted by an independent agency (Zellnet Consulting, New Jersey, NJ) using the KS-ELISPOT automatic system (Carl Zeiss, Inc. Thornwood, NY) for the quantitative analysis of the number of IFN-γ spot forming cells (SFC) for 10^5^ input of PBMCs. Responses were considered positive when the numbers of SFC with the test antigen were at least five spots above the background control values from cells cultured in the media alone.

### Cytokine multiplex assays

Cytokines were measured in the plasma samples from EDTA preserved whole blood using nonhuman Primate Cytokine kit with IL-4, IL-6, IL-10, IL-12(p40),IL-1b, MCP-1, IL-13, IL-1ra, IFN-γ- and TNF-α from Millipore Corporation (Billerica, MA) as described previously [19, 23, 25]. There is 91.4%-98.1% homology between the nucleotide sequences of squirrel monkey cytokines genes and published sequences of equivalent human and nonhuman primate genes. Plasma concentrations of cytokines were determined using the cytokine bead array (CBA) methodology according to the manufacturers’ protocols. Briefly, EDTA-preserved plasma samples were centrifuged (1200 × g for 10 minutes) and aliquots were frozen at −80°C until used. Prior to assay, once-thawed plasma samples were pre-cleared by centrifuging at 10000 × g for 5 minutes. The 96-well filter plate was washed with assay buffer for 10 min at room temperature with shaking, and 25 μL of standard or control samples were dispersed in appropriate wells. After adding 25μL of beads to each well, the plate was incubated on a rotary shaker overnight at 4°C. On the following day, after washing two times with wash buffer, the plates were incubated with detection antibody for 1hr at room temperature and then incubated with 25 μL of Streptavidin-Phycoerythin for 30 min at room temperature. After washing two times with wash buffer, 150uL of sheath fluid was added to each well and multi-analyte profiling was performed on the Bio-Plex 200 system (Luminex X MAP technology). Calibration microspheres for classification and reporter readings, as well as sheath fluid, assay and wash buffer were purchased from Bio-Rad (Hercules, CA). Acquired fluorescence data were analyzed by the Bio-Plex manager 5.0 (Bio-Rad, Hercules, CA). All steps of incubations were performed on a shaker. The concentration was calculated by the Multiplex Analyst Immunoassay Analysis Software from Millipore. The minimum detectable concentrations in pg/ml for the various cytokines are as follows: IFN- γ (2.2), and TNF- α (2.1), IL-6 (0.3), IL-10 (6.2), IL-12(P40) (1.2), IL-1β (1.2), IL-13 (5.8), IL-1ra (2.4), and MCP-1(3.1).

### Statistical analysis

For statistical analysis, samples were grouped according to untreated control, and post-treated animals from which samples were obtained. Comparisons were made between each of the post-FBZ treatment samples and the control samples using a Dunnett’s test for multiple comparisons. In order to correct for using the control data in multiple comparisons only differences with a probability of less than 0.01 were considered to be significant. All statistical analyses were conducted using GraphPad Prism^®^ version 7.01 (GraphPad Software, San Diego, California USA).

## Results

The study consisted of two study groups. The Control group consisted of fifteen untreated *Giardia-*negative animals (identified as FBZ(-) Giardia(-)) that were 3–5 year old female squirrel monkeys randomly selected from the colony for blood screening in 2015, prior to the identification of Giardiasis in the colony. Study animals included ten female squirrel monkeys, 3–5 years of age that were randomly selected from social groups known to be infected with *Giardia (identified as Giardia (+) FBZ (+)*. The presence of *Giardia* was detected via light microscopy in the pooled fecal samples and was also confirmed on individual squirrel monkeys using a Rapid Membrane Enzyme Immunoassay kit (*Giardia*/Cryptosporidium Quick Check, TechLab, Blapostcksburg, VA, USA). This method is semi quantitative (rare, few, moderate, many), but, any degree of positivity indicates infection and is promptly addressed to prevent increasing severity of infection. The infected group *Giardia (+) FBZ (+) of* animals were treated two rounds of FBZ-treatment as described in above in study group section. After four weeks of stopping of FBZ-mediated diet, blood collected at 2, 6 and 14 weeks. All subjects included in the study were healthy and in their normal social groups at the time they were sampled.

To understand the effect of FBZ on immune responses of PBMCs of squirrel monkeys, we performed detailed analyses of cell-mediated immune responses, including assays for 1) Phenotypic analysis by flow cytometry, 2) proliferation, and IFN-γ ELISPOT, in response to stimulation with mitogens (e.g., Con A, PHA, PWM, and LPS), 3) circulating levels of cytokines in plasma (e.g. IL-4, IL-6, IL-10, IL-12(p40),IL-1b, MCP-1, IL-13, IL-1ra, IFN-γ- and TNF-α), complete blood count and blood chemistry analysis before, and after FBZ treatment.

### Effect of Fenbendazole on major lymphocyte subsets in peripheral blood

**Fig 1:**
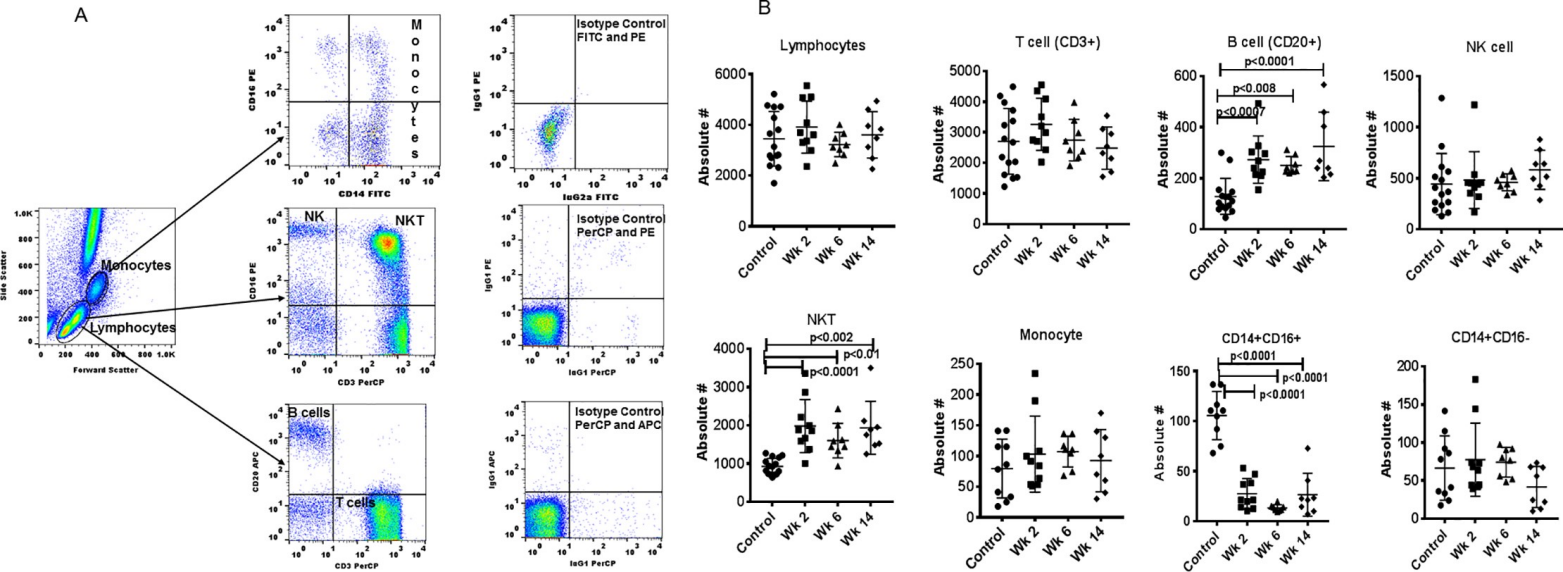
**(A)** Gating scheme for phenotype analyses of the various cell markers in the peripheral blood from a representative animal. The lymphocytes and monocytes were first gated based on forward scatter (FCS) versus side scatter (SSC), and then CD3^+^ (T cells), CD14+ (monocytes), CD3^-^CD16^+^ (NK) cells, CD3^+^CD16^+^ (NKT) cells), CD20^+^ (B cells), CD14+CD16+ and CD14+CD16- (monocytes) were positively identified by specific markers. The specificity of staining for the various markers was ascertained according to the isotype control antibody staining used for each pair of combination markers, as shown. **(B)** Effect of Fenbendazole differences in lymphocytes in squirrel monkeys. Aliquots of EDTA whole blood were stained with fluorescence-labeled antibodies to identify CD3^+^, CD14^+^, CD20^+^, and CD16^+^ cells and analyzed for cell subpopulations such as CD3+T Cells, CD20+B cells, CD16+NK cells, CD3+CD16+NKT cells and CD14 monocytes in squirrel monkeys. Monocytes (CD14) were further defined as CD14+CD16+ and CD14+CD16- monocyte. Values on the Y-axis are absolute lymphocytes cells. P values were considered statistically significant at p<0.01. FBZ (-) *Giardia* (-) group (n = 15), FBZ (+) *Giardia* group (+) week 2 (n = 10), and week 6 (n = 8) and week 14 (n = 8).

Phenotypic analysis of Monocytes, T cells, B cells, NK cells, and NKT cells from whole blood was successfully completed with flow cytometry by using the gating strategy shown in Fig 1A. We observed a significant increase in the absolute numbers of NKT (CD3+CD16+) in FBZ+ treated animals compared to controls (p<0.01) at week 2 (p<0.0001), at week 6 (p<0.0001) and at week 14 (p<0.0001) (Fig 1B). Similarly, B cells (CD20+) were also significantly higher after FBZ treatment (p<0.0007) week 2, week 6 (p<0.008) and week 14 (p<0.0001) (Fig 1B). Since *Giardia*-infection may stimulate macrophages, we further defined monocytes as CD14+CD16+ and CD14+CD16- cells and observed significant lower number of CD14+CD16+ population at week 2 (p<0.0001), week 6 (p<0.0001) and week 14 (p<0.0001) as compared to controls. No significant changes are observed in CD14+CD16- population. Analyses of the other lymphocyte subsets, T-cells (CD3^+^), monocyte (CD14^+^) and NK cell (CD16^+^) revealed no significant differences (Fig 1B).

### Effect of Fenbendazole on mitogen-induced proliferation responses

**Fig 2:**
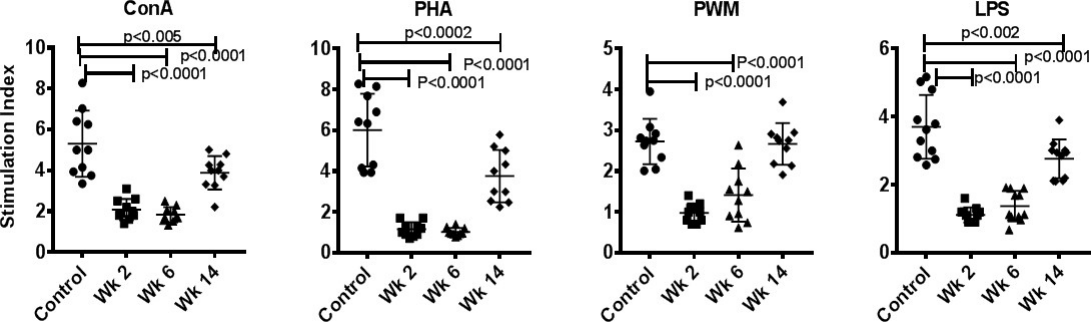
Proliferative response of PBMCs to mitogens. PBMCs that were isolated from blood samples from FBZ (-) *Giardia* (-) group (n = 15), FBZ (+) *Giardia* (+) group week 2 (n = 10), and week 6 (n = 8) and week 14 (n = 8) after FBZ treatment of the monkeys, were used for determining proliferative response to various mitogens, using the standard MTT dye reduction assay. Proliferation responses were expressed as stimulation index (SI) after blank (i.e., media only) subtraction. P values of <0.01 were considered statistically significant.

PBMCs were analyzed for proliferative responses to stimulation with Con A, PHA, PWM, and LPS. Analysis of proliferative responses show that exposure to FBZ results in a significant reduction in ConA stimulated PBMC at week 2 (p<0.0001), week 6 (p<<0.0001) and week 14 (p<0.005) in the lymphocyte proliferation after *in vitro* stimulation with PHA, Con A, PWM and LPS (Fig 2).

### Effect of Fenbendazole on mitogen-induced IFN-γ ELISPOT responses

**Fig 3:**
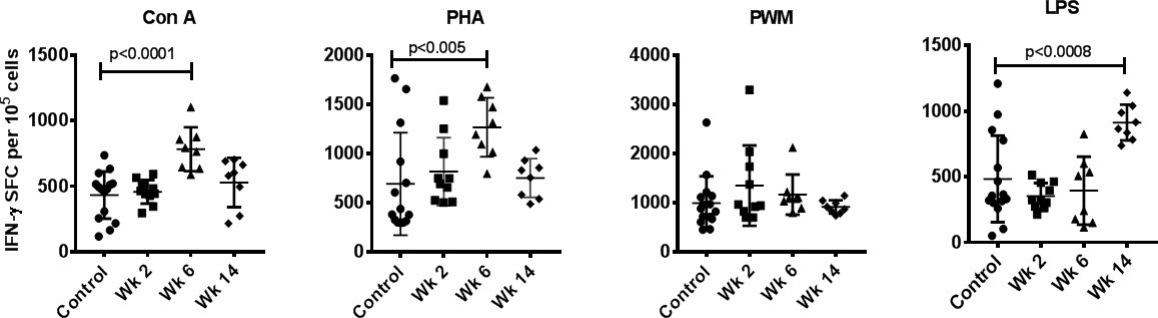
IFN-γ ELISPOT response to mitogens. PBMCs that were isolated from blood samples from FBZ (-) *Giardia* (-) group (n = 15), FBZ (+) *Giardia* (+) group week 2 (n = 10), and week 6 (n = 8) and week 14 (n = 8) after FBZ treatment of the monkeys were used in this assay. Triplicate wells of the 96-well microtiter plates, pre-coated with IFN-γ antibody were seeded with 10^5^ PBMCs, stimulated with 1 μg of each of the mitogens for 24 h at 37ºC, and then washed and stained with biotinylated secondary IFN-γ antibody. The total number of spot forming cells (SFCs) in each of the mitogen-stimulated wells were counted and adjusted to control medium as background. P values <0.01 were considered statistically significant.

PBMCs were analyzed for IFN-γ producing cells in response to stimulation with Con A, PHA, PWM, and LPS by the cytokine ELISPOT assay. Significantly higher numbers of IFN-γ secreting cells were observed at week 6 in response to Con A (p<0.0001) and PHA (p<0.001) stimulation of PBMCs from study animals compared to untreated controls. By week 14, the number of IFN-γ producing cells were not significantly different among study animals compared to controls after stimulation with Con A or PHA. (Fig 3). Stimulation with LPS revealed a significant increase in IFN-γ producing PBMCs only at week 14 (p<0.0005). No significant differences were observed for IFN-γ producing PBMCs when stimulated with PWM (Fig 3).

### Influence of Fenbendazole treatment on circulating cytokines in plasma

**Fig 4:**
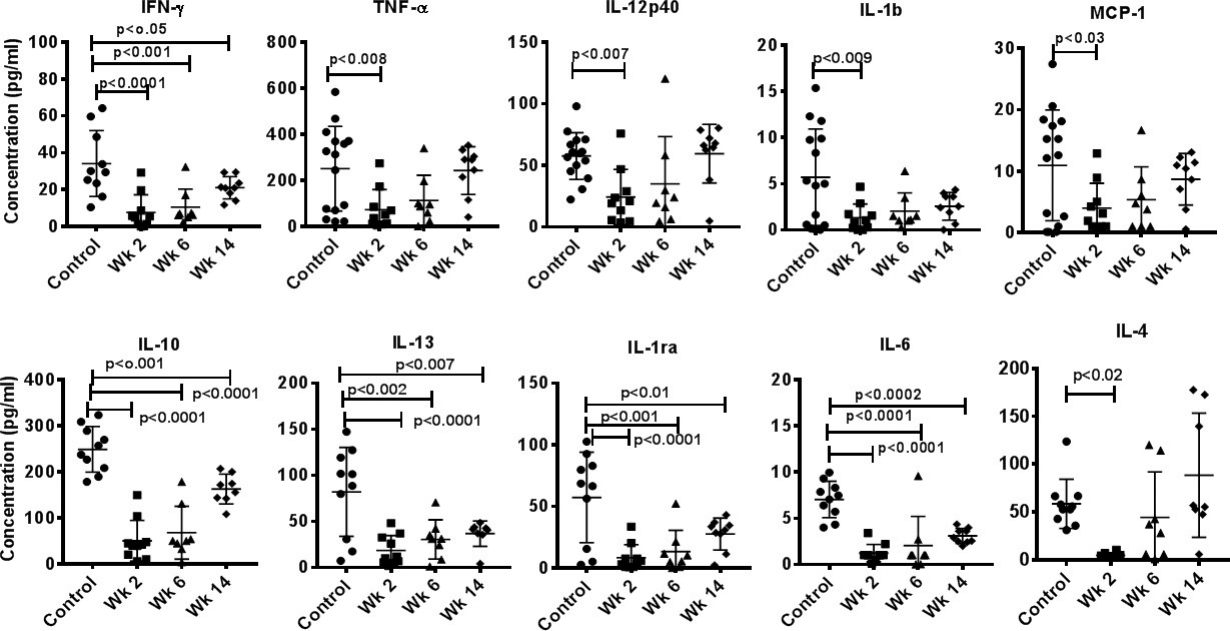
Cytokine bead array (CBA) analyses of plasma samples. In duplicate wells of the 96-well filter plate, 25 μL of plasma sample FBZ (-) *Giardia* (-) group (n = 15), FBZ (+) *Giardia* (+) group week 2 (n = 10), and week 6 (n = 8) and week 14 (n = 8), after FBZ treatment of the monkeys was incubated with 25 μL of cytokine-coupled beads overnight at 4ºC, followed by washing and staining with biotinylated detection antibody. The plates were read on BioRad 200 with use of Luminex technology, and the results were expressed as pg/mL concentration. The minimum detectable concentrations in pg/mL for IFN-γ (2.2), IL-6 (0.3), IL-10 (6.2), IL-13 (5.8), IL-12(p40) (1.2), IL-1β (1.2), IL-ra (2.4), TNF-α (2.1), IL-4 (3.1) and MCP-1 (3.1) were used for considering positive responses. P values <0.01 were considered statistically significant.

The Plasma concentrations of cytokines were determined by using a multiplex cytokine detection kit based on the Luminex technology. Comprehensive analysis of 10 different cytokines in the plasma samples from untreated control, after FBZ treatment of the monkeys showed significantly lower plasma levels of IFN γ, TNF α, IL2-40, IL-1β, IL-10, IL-13, IL-1ra, IL-6, and IL-4. Only, MCP-1 showed no effect of FBZ -treatment (Fig 4).

### Complete blood count and blood chemistry analysis

The mean total leukocyte count (WBC) for the blood collected from FBZ (+) was significantly decreased as compared to the mean, age-matched, FBZ (-) *Giardia* (-) controls. The mean WBC value of the blood collected 6 weeks after FBZ treatment (wk. 6) was significantly lower from the mean control WBC value. Similar changes in the treated samples were identified for the mean absolute monocyte, neutrophil and eosinophil counts in that all three of these values were significantly reduced as compared to their respective mean control values. The mean at wk-6 values for all three of these parameters was slightly higher than their respective mean treated values but still well below the mean control values. The absolute lymphocyte counts did not demonstrate a similar trend and the values were statistically similar between all three cohorts. At week 14, mean absolute WBC, monocyte, neutrophil and eosinophil counts were slightly higher but still well below the mean control values.

**Fig 5:**
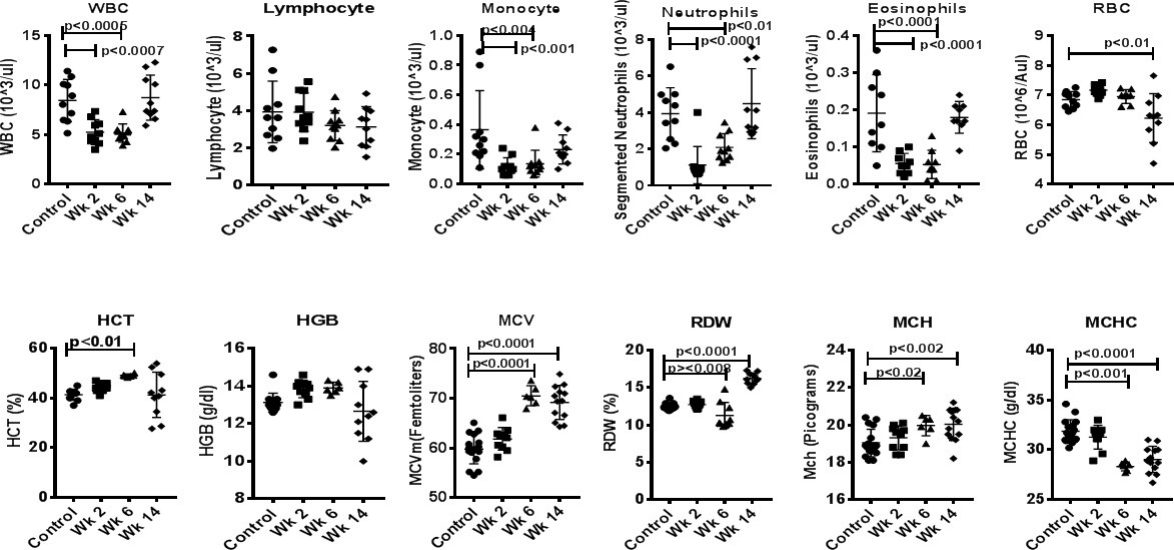
Complete blood count and blood chemistry analysis. Blood samples were taken from a peripheral vein from FBZ (-) *Giardia* (-) group (n = 15), FBZ (+) *Giardia* (+) group week 2 (n = 10), and week 6 (n = 8) and week 14 (n = 8), are analyzed for a complete blood count (Siemens Advia 120 Hematology Analyzer, Tarrytown, NY) and serum chemistry profile (Olympus AU400e® Chemistry Immuno Analyzer, Brea, CA).

With regard to the erythrocyte parameters, the HCT, HGB, MCV, and MCH mean treated values were elevated as compared to control values. This elevation was statistically significant for HCT at week 6. The mean values of HCT, HGB, MCV, and MCH at-wk-6 were higher than their respective values at week 2 and all four week6 values were significantly elevated above their respective mean control values. The MCHC of study animals was decreased compared to controls at week 6 and this effect persisted until week 14. The RBC and RDW values of study animals were elevated as compared to their respective mean control values. This change was statistically significant for RBC but not RDW. Furthermore, the RBC and RDW mean treated values were decreased as compared to the mean after wk-6 values. Here, RDW, but not RBC, was significantly lower than the mean control value. No significant changes were identified for the platelet count or platelet volumes for study animals compared to controls. As such, the data from the platelet parameters have not been included here and are not discussed further in this report. Comparisons were made between each of the FBZ-treated animals and controls using a Dunnett’s test for multiple comparisons tests and shown in Fig 5.

## Discussion

Squirrel monkeys have been an experimental host for a range of human pathogens, and are used for the assessment of vaccine candidate antigens and vaccine strategies. The present report discusses the effect of FBZ on squirrel monkey T-, B- lymphocytes, monocytes and immune effector function. This study suggested that fenbendazole may have immunomodulatory properties that are associated with altered function of mitogen specific proliferation of T-lymphocytes and IFN-γ producing lymphocytes. This observation is consistent to the immunomodulatory effect of levamisole, an anthelminthic drug used in some areas of the world, on cell-mediated immune function in dogs [26–28]. A similar observation of decreased T lymphocyte function has been described by Stankiewicz *et al*., (1995) in sheep, following exposure to Ivermectin [7]. Repeated use of the benzimidazole group showed interference with the lymphocyte responsiveness to stimulating mitogens in lambs and young sheep fed a low protein diet [2, 29, 30]. Results of similar treatments in healthy mice are not conclusive. Administration of FBZ to mice stimulated the proliferative response of T- and B-cells to non-specific polyclonal activation [31]. An opposite effect was observed by Reiss, *et al*.[13], reporting that anthelmintic treatment did not interfere with immune responses in mice as examined by induction of specific cytolytic T-lymphocytes (CTLs) *in vitro*, influenza-specific memory T cells *in vivo*, influenza-specific antibody secretion *in vivo*, or influenza-specific helper T-cells and CTLs *in vitro* [13]. FBZ has been reported to block mitosis of human lymphocytes *in vitro* [32]. In addition, FBZ treatment of sheep reduced peripheral blood lymphocyte responses to ConA and PHA [2]; this study also indicated that FBZ decreased the *in vivo* response to a particulate antigen. These studies may reflect species differences in sensitivity to FBZ and are consistent with the earlier-cited reports of FBZ induced myelosuppression in non-rodent, non-primate species. The results presented in the current study demonstrated reduction in lymphocyte activity following the administration of FBZ in *Giardia*-infected squirrel monkeys.

The immunosuppressive activity of FBZ, even for a short period, is undesirable as it may inhibit the ability of the animal to defend against pathogens. Moreover, if FBZ suppressive activity is sustained for longer periods, it may influence the results obtained from such treated animals when used in experiments, particularly those related to immune responses. While the use of FBZ is not uncommon in animal resource facilities, further evaluation of immune functionality of animals after FBZ-treatment is highly recommended before employing such animals in study.

We observed increased number of B-, and NKT lymphocytes and decreased number of monocytes expressing CD14+CD16+markers after FBZ treatment. These data are consistent with previously reported increases in natural killer (NK) cells and activated T-cells in levamisole adjuvant treatment to human cancer [33]. Modulations of T-cell maturation, as well as cytokine production, are potential mechanisms that may explain this shift in activity. This, together with a shift to Th1 cytokine production, are potential clues to its mode of action of FBZ [33–35]. Kullberg *et al*.[36], reported immune response to parasite infection in murine model resulting in down regulation of Th-1- cytokines response for IL-2 and IFN-g and this may lead to increased susceptibility to infection.

Untreated *Giardia*-infection may regulate innate and adaptive immune responses mediated by Th17 CD4+T cells and induction of Th1, Th2 and Th17 cytokines, including IL-2, IL-5, IL-6, IL-8, IL-12, IL-13, IL-23, IFN-γ and TNF-α [37, 38]. In contrast, Singer *et al* l [39] reported that in *Giardia*-infected mice deficient in production of IFN-γ, IL-4 and IL-13 did not require Th1 or the Th2 lymphocytes required for protection against *Giardia*. Singer *et a*l [39] concluded that a T-cell dependent mechanism plays an important role in protection against *Giardia*-infection but is not dependent on antibody and B cells.

In this present investigation, we observed significant reduction in circulating plasmas cytokines IFN-γ, TNF-α, IL2-(p40), IL-1β, IL-10, IL-13, and IL-ra after FBZ treatment. Similar observations were seen in mice experimentally infected with pinworms and treated with FBZ [40] T-helper (Th 2) cytokines remained attenuated after 4-week of FBZ treatment [40]. In another study in mice, distinct differences were observed in Th1- and Th2-type and T-regulatory responses during the intestinal, tissue migration and larval establishment stages of *T*. *zimbabwensis* infection [41]. There are a few reports documenting the effects of pinworms on the host hematopoiesis. Bugarski et al [42] showed significant hematopoietic alterations, characterized by increased myelopoiesis and erythropoiesis in S. *obvelata*-infected inbred CBA mice. Thus, the resultant inflammation causes an increased production in the number of eosinophils and macrophages in the host.

Reduced immune responses resulting in prolonged infection with *Giardia* in untreated cattle has been reported [43]. Babaei et al [44] showed significant increased cytokine levels in serum of symptomatic *Giardia*-infected human compared to asymptomatic and healthy controls. These studies show that both host and parasitic factors determine the outcome of infection by altering immune responses [43, 44].

The CBC data from the study suggest that FBZ treatment in squirrel monkeys results in a mild-moderate selective leukopenia, and reduced absolute monocyte, neutrophil, and eosinophil counts. The suppressive effect of treatment appears to be subsiding over time to some degree as per the results of the bloodwork obtained from at 6-week treatment cohort. Interestingly, and perhaps counterintuitively to the findings from other parts of this study, the absolute lymphocyte counts are largely unaffected by FBZ treatment. The effect of FBZ on lymphocytes is functional, therefore, and not realated to population size.

Collectively, the erythrocyte parameters suggest that FBZ treatment results in moderate erythropoiesis. In short, there is evidence of increased erythrocyte production at 2- and 6-weeks post-treatment characterized by increased numbers of large, immature red blood cells.

The origin of the effects on the monocytes, neutrophils, eosinophils and erythrocytes in this study are unknown but could be related to one of several factors. First, it is possible that the drug itself has induced the selective leukopenia and erythropoiesis identified here. Second, given the fact that lymphocyte function appears to be altered by FBZ treatment, it is possible that the selective leukopenia and erythropoiesis identified here are being mediated through alterations in cytokine production by the lymphocytes. Finally, given that we do not have access to colony samples from untreated, *Giardia-*infected animals, the possibility remains that the selective leukopenia and erythropoiesis could be directly related to the *Giardia*-infection itself.

Limitations in this study are the absence of data from *Giardia* infected untreated group of animals and inability to measure FBZ levels in the blood of animals post FBZ treatment.

## Conclusion

Based on the information presented in this report, it can be concluded that FBZ has immunosuppressive/ immunomodulatory effects on the squirrel monkey immune system. We hypothesize that FBZ-treated squirrel monkeys may not have been able to initiate an appropriate cell-mediated immune response compared to untreated squirrel monkeys; thus creating a potentially significant confounding variable in studies that require normal host immune responses. This has important ramifications when using FBZ-treated animals in studies where immunological variables are used as measurements of efficacy. Further studies of the effects of FBZ on primate immune function are critically needed.

## Abbreviations

FBZ: Fenbendazole
FACS: Fluorescence Activated Cell Sorting
ELISPOT: Enzyme-Linked ImmunoSpot
MTT: 3-(4, 5-dimethylthiazol-2-yl)-2, 5-diphenyltetrazolium bromide
PHA: Phytohaemagglutinin
Con A: Concanavalin A
IL: Interleukin
LPS: lipopolysaccharide
PWM: poke weed mitogen
